# A Web-Based Calculator for the Prediction of Severe Neurodevelopmental Impairment in Preterm Infants Using Clinical and Imaging Characteristics

**DOI:** 10.3390/children5110151

**Published:** 2018-11-14

**Authors:** Zachary A. Vesoulis, Nathalie M. El Ters, Maja Herco, Halana V. Whitehead, Amit M. Mathur

**Affiliations:** Department of Pediatrics, Division of Newborn Medicine, Washington University School of Medicine, St. Louis, MO 63110, USA; nathalie.elters@wustl.edu (N.M.E.T.); herco_m@wustl.edu (M.H.); halana@wustl.edu (H.V.W.); mathur_a@kids.wustl.edu (A.M.M.)

**Keywords:** cerebellar hemorrhage, intraventricular hemorrhage, preterm, MRI, neurodevelopment, outcome prediction, white matter injury

## Abstract

Although the most common forms of brain injury in preterm infants have been associated with adverse neurodevelopmental outcomes, existing MRI scoring systems lack specificity, do not incorporate clinical factors, and are technically challenging to perform. The objective of this study was to develop a web-based, clinically-focused prediction system which differentiates severe neurodevelopmental outcomes from normal-moderate outcomes at two years. Infants were retrospectively identified as those who were born ≤30 weeks gestation and who had MRI imaging at term-equivalent age and neurodevelopmental testing at 18–24 months. Each MRI was scored on injury in three domains (intraventricular hemorrhage, white matter injury, and cerebellar hemorrhage) and clinical factors that were strongly predictive of an outcome were investigated. A binary logistic regression model was then generated from the composite of clinical and imaging components. A total of 154 infants were included (mean gestational age = 26.1 ± 1.8 weeks, birth weight = 889.1 ± 226.2 g). The final model (imaging score + ventilator days + delivery mode + antenatal steroids + retinopathy of prematurity requiring surgery) had strong discriminatory power for severe disability (AUC = 0.850), with a PPV (positive predictive value) of 76% and an NPV (negative predictive value) of 90%. Available as a web-based tool, it can be useful for prognostication and targeting early intervention services to infants who may benefit the most from such services.

## 1. Introduction

Brain injury is a common and consequential outcome of prematurity. Preterm infants are affected by three forms of brain injury: intraventricular hemorrhage (IVH), white matter injury (WMI), and cerebellar hemorrhage (CH) [1], all of which have been associated with an increased risk of adverse neurodevelopmental outcomes [2,3,4]. Although the routine cranial ultrasound is ideal for the detection of intraventricular hemorrhage [5] and may have utility at term-equivalent age for the detection of WMI [6], the brain MRI remains the gold standard, with significantly greater sensitivity for the detection of injury [7].

However, for any imaging modality to have value, it must first meet two criteria—ease of use and reliable prediction of neurodevelopmental outcomes. Four major MRI scoring systems have been developed for preterm infants by Woodward et al. [3], Kidokoro et al. [8], Chau et al. [9], and Brouwer et al. [10]. All four systems have strong correlations with adverse outcomes, however they can be challenging to reproduce in clinical practice owing to methodology (e.g., requiring brain segmentation, diffusion tractography) or practical issues (e.g., 8–15 imaging components requiring precise measurement or volume estimation, lack of cerebellar evaluation). Furthermore, all of the systems are based on imaging alone and do no incorporate clinical factors which may be separately predictive of adverse outcomes.

Although these approaches have a high negative predictive value (NPV) for infants without evidence of injury [3,11], they have very poor positive predictive value (PPV) for those with injury, ranging between 10% and 50% [3,12]. Not surprisingly, considerable opposition from some families has developed as to the utility of the term-equivalent MRI due to the lack of precision in predicting the outcome of infants with injury [13]. This ultimately led to an AAP recommendation against the routine use of term-equivalent MRI in preterm infants [14].

To overcome these challenges, an ideal scoring system would be user-friendly, account for supratentorial and cerebellar injury, incorporate clinical factors that are linked to adverse outcomes, and differentiate infants with severe neurodevelopmental delay from those with normal-moderate outcomes with a maximal degree of precision. The objective of this study was to develop a scoring system using a cross-sectional cohort of preterm infants with term-equivalent MRI, accounting for clinical risk factors, and validate the system against formal neurodevelopmental testing at 18–24 months of age. This model was then incorporated into a web-based calculator, which will be useful for prognostication and targeting early intervention services towards infants who may benefit the most from such services.

## 2. Materials and Methods

### 2.1. Patient Selection

Infants that were included in this study represent a cross-sectional population of all preterm infants that were admitted to the NICU at St. Louis Children’s Hospital. Inclusion criteria for the cohort were preterm birth (≤30 weeks estimated gestational age [EGA]) and admission to the NICU at our institution between 1 January 2007 and 31 December 2016. Infants were excluded if there was a known congenital anomaly, if the infant died before term-equivalent age, or if he/she was lost to follow-up prior to neurodevelopmental testing. Term-equivalent brain MRI and formal neurodevelopmental testing between 18- and 24-months corrected age using the Bayley Scales of Infant and Toddler Development, 3rd edition (Bayley-III) were performed for all of the infants as a part of standard clinical care.

The study protocol was reviewed and approved by the Washington University Human Research Protection Office.

### 2.2. Data Collection

Demographic and perinatal factors that were collected included gestational age at birth, birth weight, sex, race, intrauterine growth restriction, pre-eclampsia, chorioamnionitis (diagnosed by histopathology), mode of delivery, emergent delivery, administration of antenatal steroids and/or magnesium sulfate, and the Apgar score at 5 min. Clinical characteristics included diagnosis of bronchopulmonary dysplasia (BPD, defined as the need for supplemental oxygen after 36 weeks corrected gestational age), patent ductus arteriosus (PDA) requiring ligation, ventilator days, inotropic medication administration, sepsis (positive blood culture), necrotizing enterocolitis, presence of clinically apparent seizures, and severe retinopathy of prematurity (ROP, defined as stage III disease with the need for laser ablative surgery).

### 2.3. Imaging Scoring System

All infants underwent standardized cranial ultrasound screening which consisted of scans that were performed at least once in the first three days of life, again between seven and 10 days of life, and again at 30 days of life. Of note, infants with grade III/IV IVH frequently received more cranial ultrasound exams as a result of weekly surveillance for post-hemorrhagic hydrocephalus.

All infants in the study underwent a non-sedated, non-contrast MRI scan at term equivalent age, including T1, T2, and susceptibility-weighted imaging (SWI) sequences. Magnetic resonance images were acquired by using a 3-T TIM Trio system (Siemens, Erlangen, Germany). MRI scanning included anatomic images which were obtained with an axial magnetization–prepared rapid gradient echo T1-weighted sequence (TR/TE 1500/3 milliseconds; voxel size: 1 × 0.7 × 1 mm^3^) and a turbo spin echo T2-weighted sequence (TR/TE 8600/160 milliseconds; voxel size: 1 × 1 × 1 mm^3^; echo train length: 17).

Cerebellar hemorrhage was identified as a hypointensity in the cerebellum on T2 sequences with corresponding lesion on SWI. Intraventricular hemorrhage was classified using the Papile system [5]. White matter injury was identified as hyperintensity on T1 sequences with corresponding hypointensity on T2 sequences. Diffusion-weighted imaging was not used in the scoring system. All imaging was interpreted by a clinical neuroradiologist at the time of scan who was blinded to the clinical course of the infant, and was later reviewed by one author (ZAV) to confirm the findings.

The proposed scoring system consists of three components: IVH score, WMI score, and CH score. IVH was classified as none, low-grade (grade I/II), and high-grade (grade III/IV). WMI was classified as none, isolated punctate (≤2 lesions), or multiple punctate or cystic. CH was classified as none, punctate/small (<50% of cerebellar hemisphere), or large (>50% of cerebellar hemisphere), with an additional point being given for bilateral CH.

In order to develop the scoring system, infants were first sorted by neurodevelopment outcome. Infants were divided into two groups—those with severe neurodevelopmental impairment (any one Bayley-III component greater than two standard deviations below the mean [less than or equal to 70]) and those with normal-moderate outcomes (all Bayley-III components >70). An iterative score-weighting process was conducted, starting with the “baseline” scoring system where all injury features had equal weight (e.g., 0 points for absent, 1 point for present). For each iteration, the point value of a single injury feature was increased and the sensitivity/specificity were calculated. Iterations continued for that injury feature as long as the sensitivity was increasing and specificity was not decreasing. Once the optimal weighting was identified for an injury feature, the process continued with the next injury feature. The final weighted scoring system is shown in Table 1.

### 2.4. Statistical Approach

The characteristics of infants with and without severe neurodevelopmental impairment were compared using Fisher’s Exact Test for categorical variables and the Mann-Whitney *U*-test for continuous variables. Comparisons were considered significant when *p* < 0.05. Two variations of the clinical risk score were created. The first variation (“full model”) was generated as a multivariate binary logistic regression model using parameters where *p* < 0.10 in univariate analysis and factors known to be associated with adverse neurodevelopmental outcomes in previous studies. The second variation (“slim model”) was created by entering all of the collected clinical variables into a backwards stepwise logistic regression by Akaike Information Criterion (AIC) to generate the most parsimonious model, maximizing discriminatory power while minimizing the risk of over-fitting.

A composite multivariate logistic regression model was then constructed by adding the imaging score to the clinical risk score. The variance inflation factor (VIF) was calculated for all covariates in each model to assess for excessive collinearity (defined as VIF > 5). The area under the curve (AUC) was then calculated for both models and variations to assess the degree of improvement in discrimination with the addition of the imaging score. DeLong’s test for correlated ROCs was then performed to evaluate if the improvement in AUC was statistically significant.

All statistical testing was performed using R version 3.5.1 (R Foundation for Statistical Computing, Vienna, Austria). ROC testing and plots were performed using the pROC package for R [15]. The online calculator was developed using the Shiny package for R.

## 3. Results

### 3.1. Sample Characteristics

**Figure 1:**
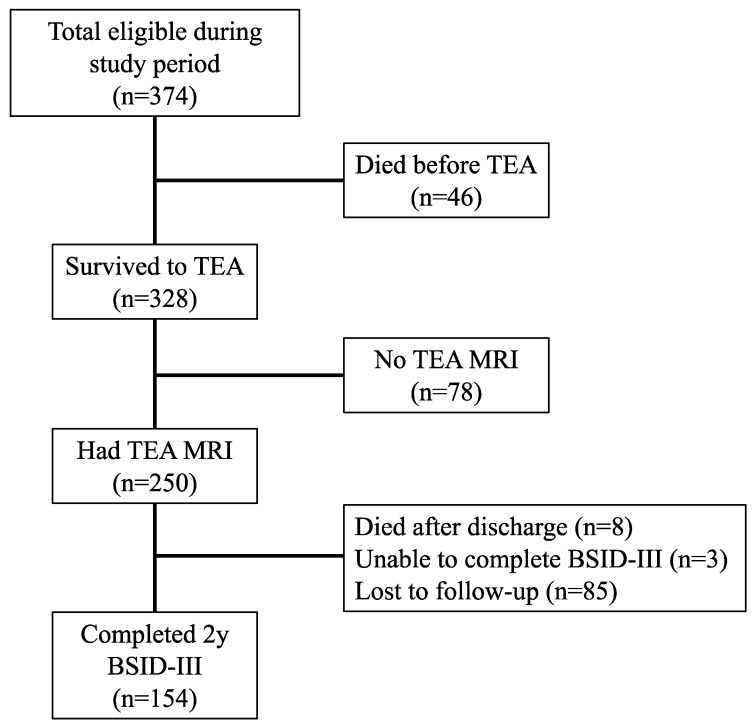
Flow diagram of patient selection.

A total of 374 eligible infants were admitted during the study period. Of those, 328/374 (88%) survived to term-equivalent age (TEA) and 250/328 (76%) underwent a brain MRI. At two years, 8/250 (3%) of the infants had died and 3/250 (1%) had such severe neurodevelopmental disability that they were not able to complete the Bayley-III. The Bayley-III was successfully completed by 154/239 (64%) of the remaining infants (Figure 1).

When comparing infants with and without severe neurodevelopmental disability, those that were affected were of lower EGA (25.6 vs. 26.2 weeks, *p* = 0.08), of lower birthweight (821.5 vs. 911.2 g, *p* = 0.07), less likely to have received antenatal steroids (71% vs. 88%, *p* = 0.02), had lower median 5-min Apgar scores (5.5 vs. 6, *p* = 0.09), received more postnatal steroids (50% vs. 31%, *p* = 0.05), had greater median ventilator days (31.5 vs. 4 days, *p* < 0.01), received more inotropic medications (50% vs. 32%, *p* = 0.05), and were more likely to have ROP requiring surgical intervention (34% vs. 18%, *p* = 0.04). See Table 2 for the complete sample characteristics.

### 3.2. Imaging Characteristics

There was a statistically significant, yet clinically irrelevant, difference in post-menstrual age (PMA) for the MRIs between severe outcome and no severe outcome (40.4 vs. 38.2 weeks, *p* = 0.02). Infants with severe outcomes had a greater median number of cranial ultrasounds (8 vs. 4, *p* < 0.01) and a higher overall incidence of brain injury of any type (90% vs. 66%, *p* < 0.01). The increased number of ultrasounds in the severe outcome group was likely the result of surveillance for post-hemorrhagic hydrocephalus in infants with high-grade intraventricular hemorrhage.

**Figure 2:**
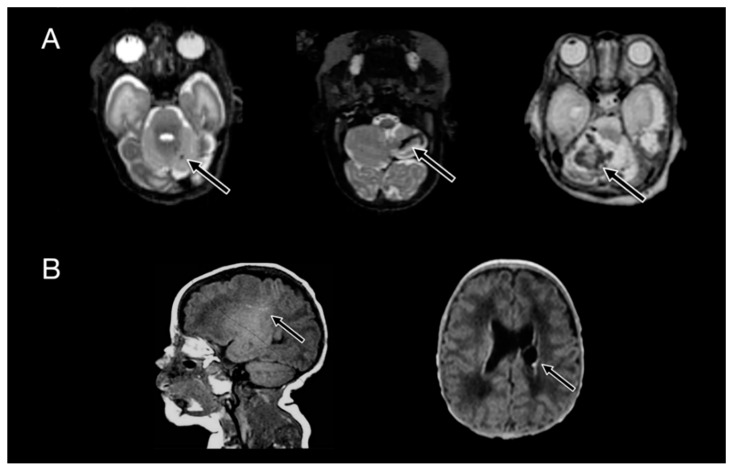
Cerebellar hemorrhage examples are shown on T2 sequences in (**A**): punctate injury is shown in the left column, small in the middle, and large in the right column. White matter injury examples are shown on T1 sequences in (**B**): punctate injury is shown at the left, while a cystic lesion is shown on the right. The primary focus of the injury is shown by the arrow.

Overall, IVH of any grade was more common in infants with severe disability (82% vs. 47%, *p* < 0.01) as was high-grade IVH (61% vs. 14%, *p* < 0.01). Cerebellar hemorrhage was more common in the severe disability group, although this difference was not statistically significant (40% vs. 22%, *p* = 0.06). Of those with cerebellar hemorrhage, infants with severe outcome more often had large hemorrhages (21% vs. 5%, *p* = 0.03) and more often had bilateral hemorrhages, although this was not significant (24% vs. 13%, *p* = 0.12). Infants with severe outcomes more often had WMI (68% vs. 32%) and were more often multiple punctate/cystic (63% vs.13, *p* < 0.01) (Table 3). Examples of CH and WMI are shown in Figure 2.

### 3.3. Clinical Logistic Regression Model

The first variation of the clinical risk score was constructed from biologically plausible and statistically-likely (defined as *p* < 0.10) factors including gestational age, birth weight, antenatal steroids, 5-min Apgar scores, postnatal steroids, ventilator days, inotrope use, and ROP requiring surgical treatment. The model R^2^ was 0.146, with a modest AUC of 0.694, and an AIC of 132.1. The second “slim” variation was generated by backwards stepwise multivariate logistic regression by AIC (ventilator days, delivery mode, antenatal steroids, and ROP requiring surgery). The model R^2^ was 0.140, an improved AUC of 0.727, and an AIC 127.8. The VIF for covariates was <5 in both variations.

### 3.4. Image Scoring System

After the iterative weighting process of the individual injury components, the mean MRI score in the severe impairment group was 8.1 ± 3.6, while the infants with normal-moderate outcomes had a mean score of 3.5 ± 2.5 (*p* < 0.01). A score threshold of >8 was 83% sensitive and 89% specific for the prediction of severe delay.

### 3.5. Combined Clinical and Imaging Logistic Regression Model

**Figure 3:**
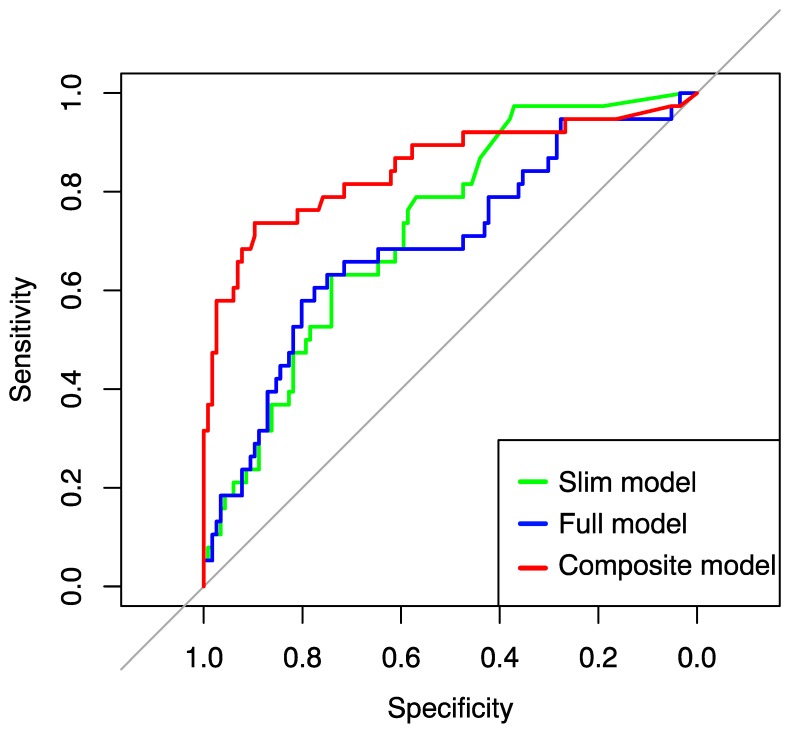
ROC curves for the “full” clinical factor model (blue), “slim” clinical factor model (green), and the composite imaging and “slim” clinical model. An interactive web-based calculator, which displays the probability ±SE of severe outcome given clinical and MRI factors, can be accessed at: https://wustl-neo.shinyapps.io/mri-calc/.

The final scoring system demonstrates the value of incorporating all aspects of preterm brain injury in an additive model in conjunction with key clinical factors. When the imaging score (CH score + IVH score + WMI score) was added to the “slim” clinical model, the composite model was strongly predictive of severe neurodevelopmental outcome. The model R^2^ was 0.458 with an excellent AUC of 0.850. The VIF for all covariates was <5. The final composite model had a PPV of 76% and an NPV of 90%. ROC curves for all models and variations are shown in Figure 3.

## 4. Discussion

In this single-center study, we have successfully developed and described a composite clinical and MRI-based scoring system which is highly predictive of severe neurodevelopmental impairment. Novel features of this scoring system are the inclusion of clinical factors in the predictive model, the use of supratentorial and cerebellar injury, and an interactive web-based risk calculator (available at https://wustl-neo.shinyapps.io/mri-calc/). This scoring system is pragmatic, clinically focused, and can be accomplished by any center which utilizes term-equivalent MRI scanning in preterm infants.

The weighting of this scoring system reveals the importance of both cerebellar and supratentorial injury in the development of severe neurodevelopmental impairment. While the impact of all three forms of brain injury has been previously described [3,5,7,16,17,18,19], a composite system using imaging and clinical factors, validated against neurodevelopmental outcomes, has not been previously developed. While cerebellar hemorrhage has long been linked to adverse outcomes, the relationship between specific radiographic features was not known. Given that cerebellar hemorrhage is tightly linked with both IVH [20] and white matter injury [21,22], it is impossible to ever untangle the magnitude of developmental impairment that is caused by each discrete form of injury. As a result of under diagnosis [23], CH is not well understood and no standardized imaging scoring system exists, although it is clear that MRI is the superior radiographic approach for detection [7]. Although other authors have reported imaging findings of CH in relationship to neurodevelopmental outcomes, there is no standardized method for the radiographic diagnosis of cerebellar hemorrhage. Correspondingly, this scoring system captures the composite impact of all three types of injury, enabling neurologists and neonatologists to provide informed prognostic detail to families beyond what had previously been available.

There are several important limitations of this study which should be considered. Despite a relatively large initial cohort, the final group of infants was smaller. For the 220 infants that were excluded, three primary factors drove this exclusion—mortality (25%), no term-equivalent MRI (35%), and loss to hospital follow-up (38%). Given that the mortality rate of infants that are diagnosed with CH may be as high as 67% [7], mortality is a notable limitation. However, as most preterm infants who die do so in the first 48 h of life [24,25], this period is far too short for the accurate assessment of other forms of brain injury (IVH, WMI) which evolve over longer periods of time. A perception amongst the clinical staff regarding the ambiguous clinical utility of the term-equivalent MRI was the predominant driving factor behind the lack of MR imaging; both a limitation of the study and further evidence of the vital need for a more predictive scoring system. Given the scope of the underlying cohort (nearly 400 infants over nearly 10 years) and the characteristics of the infants who are at greatest risk for brain injury (very preterm, severe lung disease, high mortality rates), it is challenging to identify, scan, and follow up infants in large numbers while avoiding bias as infants are excluded at each stage. However, it is reassuring that there was no difference in the basic clinical characteristics between those with and without MRI (EGA 26.1 vs. 26.4 weeks, *p* = 0.41; BW 845 vs. 948 g, *p* = 0.21; antenatal steroids 83% vs. 85%, *p* = 0.65) or those with and without two-year neurodevelopmental follow-up (EGA 26.3 vs. 26.8 weeks, *p* = 0.21; BW 919 vs. 992 g, *p* = 0.53; antenatal steroids 85% vs. 84%, *p* = 1.00).

Other limitations include the composite severe outcome that was chosen in the study design. The intent of the study was to identify, with as much accuracy as possible, which infants might have severe outcomes. While this represents a minority of the overall cohort (25%), this is an important group to identify early in order to provide early intervention services and prognostication for parents. Although infants with a high-risk of severe impairment likely can be identified on the basis of their clinical course, the models described above indicate that the combination of clinical and imaging factors provide more accurate differentiation than either alone. While reasonably large, the sample size is insufficient to predict specifically in which domain (motor, cognitive, language) the infant may face challenges, which might enable more tailored interventions. Likewise, a larger sample size would also allow for the accurate identification of infants with mild or moderate outcomes, although the smaller effect sizes would likely require a sample at least an order of magnitude larger than this current cohort.

Another important limitation in this study is the lack of information on other social determinants of health. All neonatal outcome prediction systems have a common flaw, namely the attribution of findings at term-equivalent age to differences in neurodevelopmental outcomes that are remote in time; in this case, approximately two years later. It is possible, and indeed likely, that other factors influence the outcome of these infants, including access to physical, speech and occupational therapy services, quality of nutrition, parent educational attainment, and exposure to environmental stressors (e.g., cigarette smoke, mold, unstable housing). Given the retrospective nature of this study, it was not possible to capture these variables, however they should be evaluated in future prospective studies. Related to this concern, the single-center nature of this study is also an important limitation. While the underlying NICU population is relatively diverse, there are very few infants of Hispanic and Asian descent, leading to uncertainty about model performance in these populations. This uncertainty is captured in the web-based tool and is expressed at the degree of confidence around the likelihood of severe outcomes. As with all software, this tool should be viewed as iterative in nature. Future updates might include a larger sample size or additional data from other centers.

Also of note, the rates of injury in this sample are somewhat higher than in other population-based studies [26]. It is unclear if this is the result of the relatively immature (thus, high-risk) nature of this cohort or if it is a function of center-specific characteristics. As noted by Brouwer et al. [10], there can be substantial differences in injury and outcomes between centers. We anticipate that the additional data in future studies will result in a reduction in model uncertainty.

Another possible future line of inquiry includes whether this system can be adapted to earlier MRI or non-MRI imaging. While the sum total of injury by the IVH mechanism is typically well established by the first 7–10 days of life, WMI takes much longer to evolve, with many experts recommending ultrasound surveillance at 30 days of life. While all infants in this study were term-equivalent age at the time of the scan, it is possible that this system could be used at an earlier PMA. Further investigation will be needed to validate this at early gestational ages. An alternative approach could be taken with ultrasound imaging. While this methodology is simpler and less expensive, it lacks the resolution to detect punctate cerebellar and white matter lesions [23,27], essentially eliminating the bottom end of the imaging scale. Significant adaptation would be needed to overcome these challenges.

## 5. Conclusions

In conclusion, this practical, clinically-focused predictive score, which takes cerebellar and supratentorial injury into account with clinical factors, can reliably differentiate infants with normal-moderate outcomes from those with severe delay. This approach has a substantially improved PPV compared to previous systems. Available as a web-based tool, it may be useful for prognostication and targeting early intervention services towards infants who may benefit the most from such services.

## Figures and Tables

**Table 1:** MRI Scoring System.

Cerebellar hemorrhage size	• >50% of cerebellar hemisphere—3 points
	• Punctate up to <50% of cerebellar hemisphere—1 point
	• None—0 points
Cerebellar hemorrhage laterality	• Bilateral—1 point
	• Unilateral—0 points
Intraventricular hemorrhage	• Grade III/IV—5 points
	• Grade I/II—2 points
	• No IVH (intraventricular hemorrhage)—0 points
White matter injury	• Multiple punctate (>2 lesions) or Cystic—5 points
	• Isolated punctate (≤2 lesions)—2 points
	• None—0 points

**Table 2:** Sample Characteristics.

Variable	No Severe Disability (*n* = 116)	Severe Disability (*n* = 38)	*p* Value
Gestational age, mean (SD), weeks	26.2 (1.9)	25.6 (1.6)	0.08
Birth weight, mean (SD), grams	911.2 (271.4)	821.5 (240.9)	0.07
SGA ^a^, *n* (%)	8 (7)	5 (13)	0.31
Male sex, *n* (%)	53 (46)	19 (50)	0.71
Race			
African-American	50 (43)	15 (39)	0.10
Asian	5 (4)	0 (0)	
Caucasian	61 (53)	20 (53)	
Hispanic	0 (0)	3 (8)	
Pre-eclampsia, *n* (%)	26 (22)	10 (26)	0.66
Chorioamnionitis, *n* (%)	40 (34)	11 (29)	0.56
Vaginal delivery, *n* (%)	30 (26)	11 (29)	0.83
Any antenatal steroids, *n* (%)	102 (88)	27 (71)	0.02 *
Complete antenatal steroids, *n* (%)	57 (49)	16 (42)	0.46
Antenatal magnesium sulfate, *n* (%)	75 (65)	20 (52)	0.25
5-min Apgar score, median (range)	6 (0–9)	5.5 (0–9)	0.09
Postnatal steroids, *n* (%)	36 (31)	19 (50)	0.05
Ventilator days, median (range)	4 (0–106)	31.5 (1–251)	<0.01 *
BPD ^b^ diagnosis, *n* (%)	70 (60)	27 (71)	0.25
Inotropic medication administration, *n* (%)	37 (32)	19 (50)	0.05
Culture-positive sepsis, *n* (%)	17 (15)	10 (26)	0.14
Necrotizing enterocolitis, *n* (%)	13 (11)	6 (16)	0.57
PDA ligation, *n* (%)	19 (16)	10 (26)	0.23
Clinically apparent seizures, *n* (%)	4 (3)	3 (8)	0.36
Severe ROP, *n* (%)	21 (18)	13 (34)	0.04 *
Bayley-III cognitive score, mean (SD)	91.5 (9.8)	73.3 (10.7)	<0.01 *
Bayley-III motor score, mean (SD)	89.7 (12.3)	65.5 (12.3)	<0.01 *
Bayley-III language score, mean (SD)	89.7 (10.2)	69.9 (13.3)	<0.01 *

^a^ Defined as birth weight< 10th centile. ^b^ Defined as the need for supplemental oxygen past 36 weeks post-menstrual age. Statistically significant associations denoted with an asterisk.

**Table 3:** Imaging Characteristics.

Characteristic	No Severe Disability (*n* = 116)	Severe Disability (*n* = 38)	*p* Value
Any IVH, *n* (%)	55 (47)	31 (82)	<0.01 *
Grade III/IV IVH, *n* (%)	16 (14)	23 (61)	<0.01 *
Any cerebellar hemorrhage, *n* (%)	26 (22)	15 (40)	0.06
Cerebellar hemorrhage size			
Large, *n* (%)	6 (5)	8 (21)	0.03 *
Small, *n* (%)	12 (10)	5 (13)	
Punctate, *n* (%)	8 (7)	2 (5)	
None, *n* (%)	90 (78)	23 (61)	
Bilateral cerebellar hemorrhage, *n* (%)	15 (13)	9 (24)	0.12
WMI (white matter injury)			
Multiple punctate or cystic, *n* (%)	15 (13)	24 (63)	<0.01 *
Isolated punctate, *n* (%)	22 (19)	2 (5)	
None, *n* (%)	79 (68)	12 (32)	
Any brain injury, *n* (%)	77 (66)	34 (90)	<0.01 *
Number of head ultrasounds, median (range)	4 (0–28)	8 (2–38)	<0.01 *
PMA at MRI	38.2 (2.6)	40.4 (5.7)	0.02 *

Statistically significant associations denoted with an asterisk.
